# Humoral and cellular immune responses after COVID-19 vaccination of lung transplant recipients and patients on the waiting list: a 6-month follow-up

**DOI:** 10.3389/fimmu.2023.1254659

**Published:** 2024-01-04

**Authors:** Rogier A. S. Hoek, Siqi Liu, Corine H. GeurtsvanKessel, Erik A. M. Verschuuren, Judith M. Vonk, Merel E. Hellemons, Mirjam Kool, Nynke Wijbenga, Susanne Bogers, Sandra Scherbeijn, Sharona Rugebregt, Johanna P. van Gemert, Willie N. Steenhuis, Hubert G. M. Niesters, Debbie van Baarle, Rory D. de Vries, Coretta Van Leer Buter

**Affiliations:** ^1^ Department of Pulmonary Medicine, Erasmus Medical Center (MC) Transplant Institute, University Medical Center Rotterdam, Rotterdam, Netherlands; ^2^ Department of Rheumatology and Clinical Immunology, University of Groningen, University Medical Center Groningen, Groningen, Netherlands; ^3^ Department of Viroscience, Erasmus Medical Center, Rotterdam, Netherlands; ^4^ Department of Pulmonary Diseases, University of Groningen, University Medical Center Groningen, Groningen, Netherlands; ^5^ Department of Epidemiology and Groningen Research Institute for Asthma and Chronic Obstructive Pulmonary Disease (COPD) (GRIAC), University of Groningen, University Medical Center Groningen, Groningen, Netherlands; ^6^ Department of Medical Microbiology, University of Groningen, University Medical Center Groningen, Groningen, Netherlands; ^7^ Center for Infectious Disease Control, National Institute for Public Health and the Environment, Bilthoven, Netherlands

**Keywords:** cellular responses, humoral responses, antibody decay, cellular decay, lung transplantation, waitlist

## Abstract

**Background:**

Data on cellular response and the decay of antibodies and T cells in time are scarce in lung transplant recipients (LTRs). Additionally, the development and durability of humoral and cellular immune responses have not been investigated in patients on the waitlist for lung transplantation (WLs). Here, we report our 6-month follow-up of humoral and cellular immune responses of LTRs and WLs, compared with controls.

**Methods:**

Humoral responses to two doses of the mRNA-1273 vaccination were assessed by determining spike (S)-specific IgG antibodies and neutralizing antibodies. Cellular responses were investigated by interferon gamma (IFN-γ) release assay (IGRA) and IFN-γ ELISpot assay at 28 days and 6 months after the second vaccination.

**Results:**

In LTRs, the level of antibodies and T-cell responses was significantly lower at 28 days after the second vaccination. Also, WLs had lower antibody titers and lower T-cell responses compared with controls. Six months after the second vaccination, all groups showed a decrease in antibody titers and T-cell responses. In WLs, the rate of decline of neutralizing antibodies and T-cell responses was significantly higher than in controls.

**Conclusion:**

Our results show that humoral and cellular responses in LTRs, if they develop, decrease at rates comparable with controls. In contrast, the inferior cellular responses and the rapid decay of both humoral and cellular responses in the WL groups imply that WLs may not be protected adequately by two vaccinations and repeat boostering may be necessary to induce protection that lasts beyond the months immediately post-transplantation.

## Introduction

Solid organ transplant recipients (SOTRs), particularly lung transplant recipients (LTRs), are at increased risk for severe coronavirus disease-2019 (COVID-19) because of their chronic immunosuppressive state but also because of infection of the allograft itself (1–7). LTRs have reduced immune responses after COVID-19 vaccination compared with controls, with antibodies developing in 0%–64% of patients (8–10). Cellular responses have been studied to a lesser extent, but severe acute respiratory syndrome coronavirus-2 (SARS-CoV-2)-specific T lymphocytes can be detected in 2%–56% of LTRs after the first two vaccinations, which is lower compared with controls (11). Virus-specific T cells can nevertheless be present without detectable antibodies (10–13). Studies into the durability of antibodies indicate that healthy controls lose 90% of antibodies in 6 months after the primary vaccination regimen. Data regarding antibody decay in SOTR are conflicting, as different vaccination schedules have been followed in these studies (12–16). To our knowledge, there are no data reporting the durability of cellular responses in LTRs over time, but studies in other types of organ recipients have concluded that cellular responses 6 months after vaccination are significantly lower compared with healthy individuals (16–18).

Waiting list patients (WLs) for lung transplantation are also at risk for developing severe COVID-19. No studies to date have investigated humoral and cellular immune responses to vaccination in this group. A study on kidney transplant recipients showed that patients on the waiting list responded well to vaccination (19). However, kidney transplant recipients (KTRs) originally vaccinated while on the waitlist (pre-transplantation) did not respond to a booster vaccination given post-transplantation (20). These studies emphasize the need to achieve optimal protection of WLs prior to the commencement of antirejection therapy after transplantation. With no data on the induction and durability of immune responses of WLs for lung transplantation and with limited evidence about the durability of immune responses in LTR, here, we investigated these two groups in a 6-month follow-up study. We describe the induction and kinetics of humoral and cellular immune responses in these groups by measuring binding antibodies, neutralizing antibodies, and cellular responses by performing interferon gamma (IFN-γ) release and enzyme-linked immunospot (ELISpot) assays.

## Materials and methods

### Study participants

**Figure 1:**
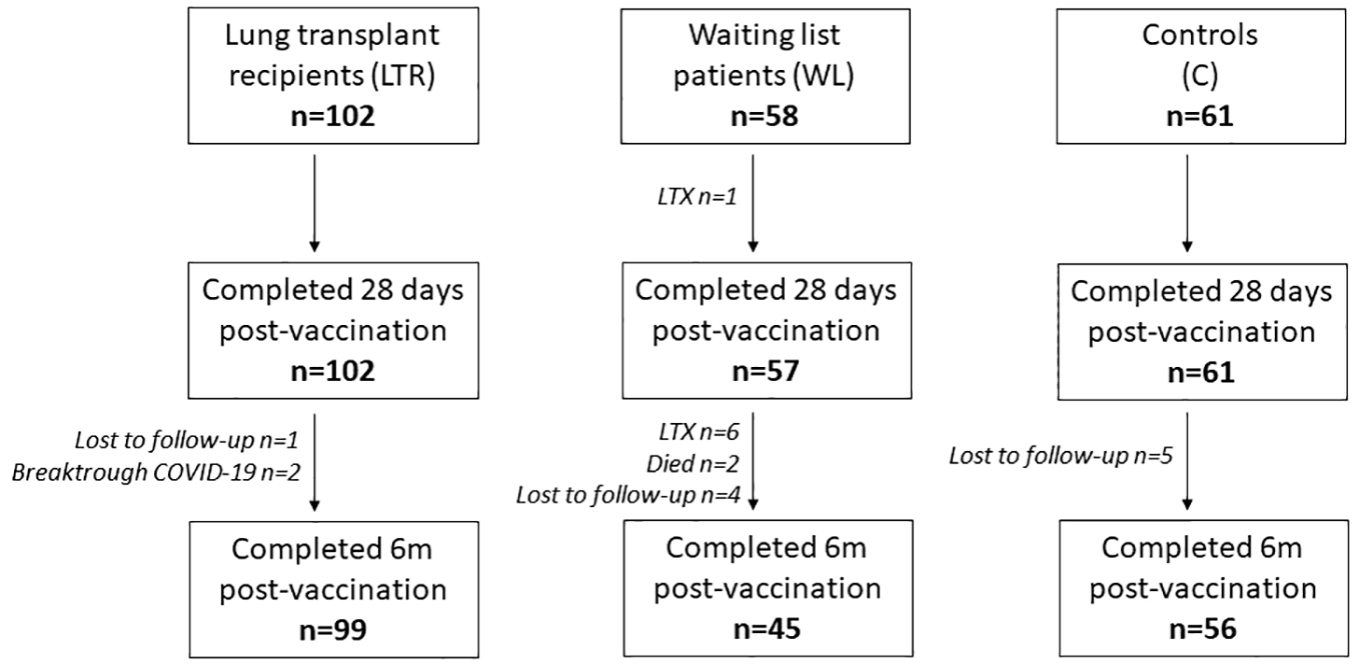
Number of participants during the study at each time point. LTRs, lung transplant recipients; 6m, 6 months.

Between 23 February and 21 March 2021, 221 participants were enrolled in the COVALENT study at the Erasmus Medical Center in Rotterdam and the University Medical Center in Groningen (LTRs, *n* = 102; WLs, *n* = 58; controls, *n* = 61) to detect humoral and cellular immune responses at baseline (T0), 28 days after first vaccination (T1), 28 days after second vaccination (T2), and 6 months after second vaccination (T3). Control participants consisted of controls in the RECOVAC study, a study investigating the immunogenicity of COVID-19 vaccines in patients with renal failure (21). Controls did not receive immunosuppressants and had no history of renal failure. Participants with a past SARS-CoV-2 infection were excluded, as were LTRs with rejection episodes less than 6 months ago. During the study period, participants who tested positive for SARS-CoV-2 and WLs who received transplantation were excluded from further analysis. A limited number of participants was lost to follow-up for unknown reasons (**Figure 1**). At T2, 220 participants were still included in the study (102 LTRs, 57 WLs, and 61 controls); at T3, 200 participants were still included (99 LTRs, 45 WLs, and 56 controls).

### Procedures

All participants received the mRNA-1273 vaccine by intramuscular injection in February and March 2021 with an interval of 28 days, according to the manufacturer’s instructions. Blood samples were collected at T0, T1, T2, and T3. Cell counts and routine chemistry were determined, as well as demographic data, lung function, antirejection medication (including trough levels), age, sex, transplantation type and date, and reason for transplantation.

### Antibody response

Spike (S)-specific IgG binding antibodies in serum were measured using the Liaison platform as previously described (DiaSorin, France) (9). An antibody titer of >33.8 BAU/ml was considered positive. Nucleocapsid (N)-specific antibodies were measured at each time point using the Alinity I platform (Abbott, Chicago, IL, USA), to exclude SARS-CoV-2 infections in LTRs as well as in the control group (9). Participants with suspected SARS-CoV-2 infections prior to vaccination or during the follow-up period were excluded from further analysis. Neutralizing antibodies against the ancestral SARS-CoV-2 were assessed as described previously with a plaque reduction neutralization test (PRNT) in VeroE6 cells in participants positive for the presence of binding antibodies. Results are expressed as geometric titers (GMT) of virus-neutralizing antibodies and a PRNT50 >10 was considered positive (22).

### Interferon gamma release assay

An IFN-γ release assay (IGRA) was used to measure virus-specific T-cell responses after stimulation of whole blood with antigen formulations from the spike (S) protein according to the manufacturer’s instructions (QuantiFERON, Qiagen, Hilden, Germany). Values of ≥0.150 were considered positive, and the measured values in response to Ag2 stimulation expressed in IU/ml were used to compare secreted IFN-γ levels after subtraction of the negative control values as interpolated from a standard calibration curve (18).

### IFN-γ ELISpot assay

Peripheral blood mononuclear cells (PBMCs) were isolated by density gradient centrifugation using Ficoll-Paque Plus (GE Healthcare, Freiburg, Germany) and Sepmate-50 tubes (Stemcell, Cologne, Germany) according to standard protocols (22). SARS-CoV-2 S-specific IFN-γ-producing T cells were measured by IFN-γ ELISpot assay as previously described (23). In brief, PBMCs were stimulated overnight with two pools of overlapping 15-mer peptides (JPT Peptide Technologies, Berlin, Germany) covering the full-length SARS-CoV-2 S protein. All samples were measured in triplicate, and spots were counted, using an AID ELISpot Reader (Autoimmun Diagnostika, Strassberg, Germany). Total S-specific responses were determined by summing the responses of the two peptide pools. A response at T2 was defined as a two-fold increase from baseline and higher than 50 spot-forming colonies (SFC)/10^6^ PBMCs.

### TTV PCR

A quantitative Torque Teno virus (TTV) PCR was carried out on samples obtained at baseline, as the plasma levels of TTV genomes are thought to be a reflection of the immune function (24). A quantitative PCR was performed using the R-Gene method as reported previously (BioMérieux, France) (9).

### Statistical analysis

SPSS version 28 was used for statistical analysis. Continuous data are presented as mean (± standard deviation) in case of normal distribution, or median (± interquartile range, IQR) in case of non-normal distribution. Categorical data are presented as numbers (percentages). Vaccine responses based on the four different outcome measures were compared between the groups at all-time points. Differences in percentage of responders were tested using the chi-square test, and differences in measured levels were compared using the Mann–Whitney *U*-test test (for non-normally distributed data) and *t*-test (for normally distributed data). To investigate if baseline factors [gender, age, TTV, >100 months since transplantation, use of azathioprine (AZA), use of mycophenolate mofetil (MMF), use of steroids] were associated with vaccine response, logistic regression analyses were performed in each investigated group separately. First, univariate associations were tested and all factors with a *p*-value <0.15 were entered into a multivariate model. Since the use of AZA and MMF is highly correlated, only the medication with the lowest *p*-value in the univariate model was entered in the multivariate model. The correlation between IgG antibody levels and neutralizing antibodies was determined using Spearman’s rho test.

To investigate the change in vaccine response over time, the percentage change between 28 days and 6 months after the second vaccination was calculated in patients with a positive response at 28 days after the second vaccination. The percentage change in the measured levels of vaccine response within each group was compared using the Wilcoxon signed-rank test, and differences in the percentage change over time between the groups were tested using the Mann–Whitney *U*-test. Two-sided *p*-values were reported; a *p*-value <0.05 was considered to be significant. Figures were created with the software GraphPad Prism version 5.00 (GraphPad Software, San Diego, CA, USA).

## Results

### Characteristics of the study population

Demographic and clinical characteristics of LTRs and WLs are reported in **Table 1**. Time since transplantation was <100 months for 73 LTRs (71.6%) and >100 months for 29 LTRs (28.4%). Obstructive lung disease was the most common reason for transplantation in LTRs and WLs. A total of 98 (96.1%) LTRs were on a tacrolimus-based treatment regime with MMF (90, 88.2%). In WLs, immunosuppressants were administered to 23 participants (40%), of which 19 received corticosteroids (32.8%).

**Table 1:** Clinical characteristics of lung transplant recipients (LTRs) and waitlist patients (WLs).

	LTRs	Waiting list
*N*	102	58
Female, *n* (%)	48 (47.1)	33 (56.9)
Caucasian, *n* (%)	96 (94.1)	55 (94.8)
Age, years (median (IQR))	60 (49–66)	59 (53–63)
BMI, kg/m^2^ (mean ± SD)	24.6 ± 4.6	25.1 ± 3.2
Laboratory, median (IQR)		
Leukocytes, 10^9^/L	7.4 (5.8–8.9), *n* = 97	7.8 (6.6–9.8), *n* = 18
Lymphocytes, 10^9^/L	1.4 (0.9–2.1), *n* = 55	1.8 (1.4–2.1), *n* = 8
TTV		
% positive (TTV >1)	96 (94.1)	27 (47.4)
TTV in positive subjects	5.4 ± 1.9	3.0 ± 1.0
Reason for LoTX, *n* (%)		
Obstructive lung disease	48 (47.1)	45 (77.6)
Suppurative lung disease	18 (17.6)	2 (3.4)
Restrictive lung diseases	28 (27.5)	10 (17.2)
Disease of the pulmonary circulation	7 (6.9)	1 (1.7)
Unknown	1 (1.0)	
Time since Tx, *n* (%)		
<100 months	73 (71.6)	
>100 months	29 (28.4)	
Immunosuppressive treatment, *n* (%)		
Corticosteroids	94 (92.2)	19 (32.8)
Mycophenolate mofetil (MMF)	90 (88.2)	2 (3.4)
Azathioprine	9 (8.8)	1 (1.7)
Tacrolimus	98 (96.1)	0 (0.0)
mTOR inhibitor	15 (14.7)	0 (0.0)
Rituximab	2 (2.0)	1 (1.7)
Cyclosporine	2 (2.0)	0 (0.0)
Other		
MMF–tacrolimus–prednisolone	83 (81.4)	0 (0.0)
AZA–tacrolimus–prednisolone	8 (7.8)	0 (0.0)
Number of immunosuppressive agents	3 (3-3)	0 (0-1)
Comorbidities, *n* (%)		
Hypertension	60 (58.8)	18 (31.0)
Diabetes mellitus	47 (46.1)	3 (5.2)
Coronary artery disease	10 (9.8)	3 (5.2)
Chronic kidney disease	26 (25.5)	0 (0.0)

### SARS-CoV-2 S1-specific IgG antibody responses

**Figure 2:**
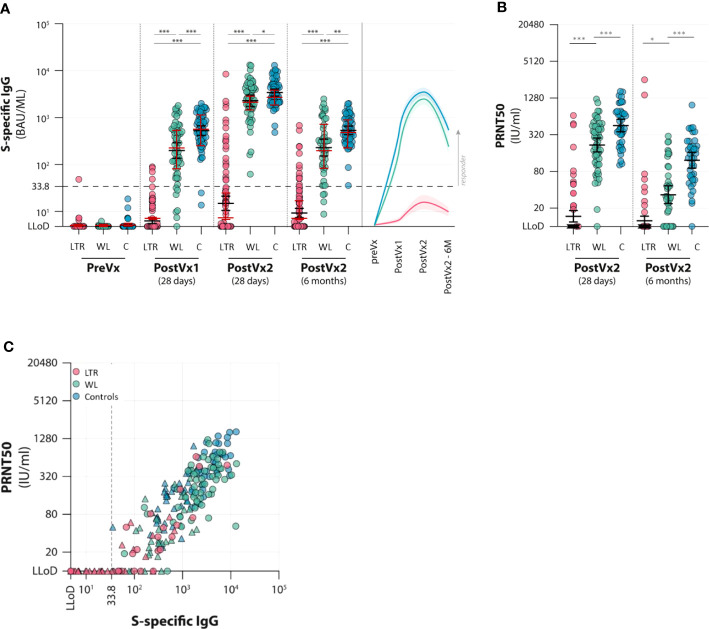
Antibody responses to two vaccinations with the mRNA-1273 vaccine in lung transplant recipients (LTRs) (red dots), waitlist patients (WLs) (green dots), and controls **(C)** (blue dots). Three time points were assessed: pre-vaccination (preVx), 28 days after the first vaccination (PostVx1), 28 days after the second vaccination [PostVx2 (28 days)], and 6 months after the second vaccination [PostVx2 (6 months)]. **(A)** Titer of S-specific IgG (BAU/ml). Black error bars: geometric mean titers (GMTs) (+/− SD), red error bars: median (+/− IQR). *p = 0.023, **p = 0.0011, ***p < 0.0001. The dotted line represents the cutoff for the positive test according to the manufacturer. (Precise *p*-values are reported in the **Supplementary Data**). **(B)** GMT of neutralizing antibodies (IU/ml). *p = 0.065, ***p < 0.0001. At 6 months after vaccination, GMTs decreased significantly in all three groups (*p* = 0.021 in LTRs; in WLs and controls *p* < 0.0001). The percentage decrease of GMT between the two time points is significantly higher in WLs (*p* < 0.0001). **(C)** Spike-specific IgG levels (*x*-axis) versus neutralizing antibodies (PRNT50) (*y*-axis) in three patient groups (LTRs, WLs, and controls, determined at 28 days after the second vaccination (dots) and after 6 months (triangles). The graph shows that for participants who develop antibodies, neutralizing antibodies correlate with IgG levels (Rho in LTRs 0.802, in WLs 0.593, and controls 0.704).

At T1, 28 days after the first vaccination, only 4 of 102 LTRs (4%) had detectable binding antibodies (>33.8 BAU/ml) with a median titer in responders of 77 BAU/ml (IQR 54–88 BAU/ml), compared with 51 of 58 (88%) WLs and 59 of 61 controls (97%). WL participants (both responders and non-responders) had a median titer of 249 BAU/ml (IQR 84–565 BAU/ml), which was significantly lower than controls who had a median titer of 562 BAU/ml (IQR 333–1,075 BAU/ml). At T2, 28 days after the second vaccination, the number of LTR responders increased to 21 of 102 (21%), with median antibody titer in responders of 311 BAU/ml (IQR 96–829), which was still inferior to the other groups. All WL participants and controls developed antibodies, with a median titer of 2,520 BAU/ml and 3,220 BAU/ml, respectively. WLs had significantly lower antibody titers than controls (*p* = 0.02). Antibody levels decreased over time in all groups; at T3, 6 months after the second vaccination, only 13 of the 21 initial responders (62%) of the LTR group still had detectable antibodies (median 85 BAU/ml, IQR 50–242), representing a 71% median titer decrease in previous responders. At this time point, 40 of 44 (91%) of WLs still had antibodies (median 267, IQR 90–753 BAU/ml), compared with all 55 controls (median 465 BAU/ml, IQR 309–951 BAU/ml), indicating that WLs had significantly lower IgG levels compared with controls, but the rate of decline was similar (**Figure 2A**; **Supplementary Table 1**).

### Neutralizing antibodies

Neutralizing antibodies were determined at T2 and T3 in participants who had a binding antibody concentration >33.8 BAU/ml at T2 (**Figure 2B**). Neutralizing antibodies were detected in 15 of 21 LTRs (71%), with a GMT in the LTR− group of 40.4. At T3, only seven LTRs were positive, and the GMT in the LTR group was 18.8. Significant differences between WL and controls were observed at both time points: at T2, all but one (98%) WL had detectable neutralizing antibodies, as this group had a GMT of 217.3, which was significantly lower than controls, who all had detectable antibodies, with a GMT of 453.8 (*p* < 0.001). At T3, 28 (72%) WLs still had neutralizing antibodies with a GMT of 33.5 versus a GMT of 122.1 in controls (*p* < 0.0001). The rate of neutralizing antibody decline was higher in WLs showing an 89% decrease at T3, versus a 75% decrease in controls (**Figure 2B**; **Supplementary Table 2**).

The level of neutralizing antibodies in each group correlated significantly with the titer of S-specific IgG levels at T2 in all three groups (Rho in LTRs 0.802, in WLs 0.593, and in controls 0.704) (**Figure 2C**).

### SARS-CoV-2-specific T-cell response

**Figure 3:**
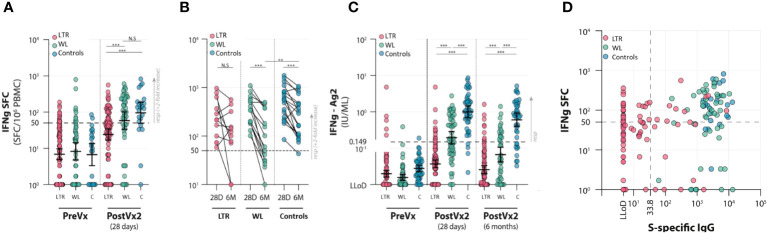
Cellular responses to two vaccinations with the mRNA-1273 vaccine in lung transplant recipients (LTRs) (red dots), waitlist patients (WLs) (green dots), and controls **(C)** (blue dots). Three time points were assessed: pre-vaccination (PreVx), 28 days after the second vaccination [PostVx2 (28 days)], and 6 months [PostVx2 (6 months)] after the second vaccination. The dotted lines represent the cutoff for the positive results. (Precise *p*-values are reported in the **Supplementary Data**). **(A)** ELISpot results showing the number of IFN-γ SFC/10^6^ PBMCs pre-vaccination and at 28 days after the second vaccination. ***p < 0.0001; N.S., not significant. (Precise *p*-values are reported in the **Supplementary Data**). **(B)** ELISpot results showing the contraction of cellular responses in time between 28 days and 6 months after the second vaccination. **p = 0.027, ***p < 0.0001; N.S., not significant. (Precise *p*-values are reported in the **Supplementary Data**). **(C)** IFNγ-release assay (IGRA) showing inferior responses in the LTR group. ***p < 0.0001. The decline in responses in the WL group between 28 days and 6 months after vaccination is disproportionately large. (Precise *p*-values are reported in the **Supplementary Data**). **(D)** Correlation between IFN-γ SFC/10^6^ PBMCs in lung transplant recipients (LTRs) (red dots), waitlist patients (WLs) (green dots), and controls (Controls) (blue dots). SFC, spot-forming cells; LLoD, lower limit of detection. The dotted lines indicate the cutoff for positive results. In WLs and controls, all participants were serological responders; therefore, two groups are formed based on cellular responses. In LTRs, humoral and cellular responses do not correlate at 28 days after the second vaccination, as four groups are distinguishable—right upper quadrant: individuals with both humoral and cellular responses (7.4% of LTRs, 53.2% of WLs, 78.3% of controls); right lower quadrant: individuals with humoral but no measurable cellular response (12.6% of LTRs, 46.8% of WLs, 21.7% of controls); left upper quadrant: individuals with measurable cellular responses but no humoral response (only LTRs in this category: 22.1% of LTRs); left lower quadrant: individuals with no humoral and no measurable cellular response (only LTRs in this category: 57.9% of LTRs).

S-specific T-cell responses were determined by IFN-γ ELISpot as well as interferon release assay (IGRA). Both assays indicated a significant difference between LTRs and the other two groups. In LTRs, the median number of SFC/10^6^ PBMCs was 38 (IQR 10–75) at T2, which was significantly lower than both WLs and controls. In WLs (*n* = 49), the number of SFC/10^6^ PBMCs was 100 (IQR 33–243) at T2. This was comparable to controls (*n* = 23), who showed 132 SFC/106 PBMCs (IQR 55–193) at T2 (**Figure 3A**). There were significantly fewer responders, defined as having ≥50 SFC/10^6^ PBMCs at 28 days, with a minimum of two-fold increase compared with pre-vaccination among LTRs (29.5%) than in the other groups [55.1% responders among WLs (*p* < 0.001) and 78.3% in controls (*p* < 0.01)] (**Supplementary Table S3**).

To determine the decay of spike-specific T-cell responses in time, IFN-γ ELISpot was performed at T3, 6 months post-vaccination in the subgroup of participants who displayed good responses at T2 and from whom sufficient material was present to perform the paired assays; 12 LTRs, 15 WLs, and 19 controls were included. In LTRs, the median number of SFC/10^6^ PBMCs decreased by 37% from 216 (90–348) at T2 to 133 (20–285) at T3, whereas in WLs, the median decrease was 74% from 342 (192–572) to 60 (30–376) SFC/10^6^ PBMCs. The reduction in controls was 49% from 573 (250–832) to 293 (103–452) SFC/10^6^ PBMCs, indicating that the decay of SARS-CoV-2-specific T cells in WLs was significantly faster than controls (*p* = 0.025) (**Figure 3B**; **Supplementary Table 3**).

A positive IGRA response of ≥0.15 IU/ml was found in 12 of 100 (12%) LTRs at 28 days post-vaccination, compared with 30 of 55 (54.5%) WLs (*p* < 0.001) and 44 of 46 (91.3%) controls (*p* < 0.001). At 6 months, 9 of 91 tested LTRs still had a positive IGRA (10%), compared with 16 of 40 (40%) WLs and 32 of 41 controls (78%) (*p* < 0.001) (**Figure 3C**). A reduced IFN-γ production upon stimulation with S peptides was observed in all groups between 28 days and 6 months after vaccination. This decrease was not significant in LTRs, but in WLs, the median decrease in responders was 71.4% (*p* < 0.001). The median decrease in controls was 40.2% (*p* < 0.001). The IFN-γ concentration declined significantly faster in WLs compared with controls (*p* = 0.040), reflecting what was observed in the ELISpot assay.

### Correlation between serology and cellular response

At 28 days post-vaccination, we investigated whether antibody levels correlated with the cellular responses measured by IFN-γ ELISpot. Serological responses did not correlate with cellular responses in LTRs, WLs, and controls separately (*p* = 0.21), but four clear groups could be identified. Of the 21 serological responders (S1-specific IgG >33.8 BAU/ml) in the LTRs, 19 were measured by IFN-γ ELISpot, seven of which were responders. IFN-γ ELISpot was performed in 76 serological non-responders, of which 21 had virus-specific T-cell responses. Of the LTRs, 7.4% were serology+/T cell+, 12.6% were serology+/T cell−, 22.1% were serology−/T cell+, and 57.9% were serology−/T cell– (**Figure 3D**).

In the WL and control groups, all participants were serological responders. There were 53.2% of WLs and 78.3% of controls who were serology+/T cell+, and 46.8% of WLs and 21.7% of controls were serology+/T cell− (**Figure 3D**).

### Association between baseline characteristics and responses

We previously reported that LTRs with superior serological responses at T2, 28 days after the second vaccine, were younger, used MMF less frequently, and had lower TTV plasma levels (9). At that time point, time since transplantation was not associated with serological response. In contrast, at T3, time since transplantation of >100 months correlated with the persistence of antibodies, but the TTV level did not. Younger LTRs were more likely to have antibodies at T3 (**Supplementary Table 5**), but age was not a significant factor in the presence of neutralizing antibodies at T2 and T3 (**Supplementary Table 6**). Furthermore, a higher prevalence of cellular responses of LTRs at T2 was associated with time since transplantation of >100 months when measured by IFN-γ ELISpot (**Supplementary Table 7**). In WLs, the use of corticosteroids was associated with a lower prevalence of a cellular response at 6 months after the second vaccination when measured by IGRA (*p* = 0.024) (**Supplementary Table 8**).

## Discussion

Inducing SARS-CoV-2-specific immune responses by vaccination has proven to be challenging in SOTRs, especially in LTRs (14, 24). We previously showed that only a small percentage of the LTRs develop antibodies following two vaccinations (9). In this study, we additionally show that T-cell responses are strongly reduced in LTRs after two vaccinations, compared with controls. Six months after the second vaccination, T-cell responses and antibodies decreased to undetectable levels in most LTRs. Interestingly, the antibodies and T-cell responses did not wane significantly faster in LTRs than in controls.

Few studies have investigated the induction of T-cell responses in LTRs, and to our knowledge, no studies exist that investigated the durability of T-cell responses in LTRs specifically. Most immune response and decay studies after COVID-19 vaccination included KTRs and showed that antibody levels decrease significantly over time. T-cell responses also decrease in time, in controls as well as in immunocompromised populations, but compared with antibodies, they are relatively stable (17, 18, 25). As more evidence points toward the importance of cellular responses in protection against severe COVID-19 (26–29), a greater understanding of how responses of different T-cell populations are induced and maintained is essential for optimizing vaccination strategies. Additionally, within SOTR groups, there are still insufficient data on the factors predisposing to poor immune responses. We and several other groups previously reported that for LTRs of older age, the use of MMF and higher TTV loads were associated with low or absent humoral responses 28 days after vaccination (9, 30, 31). TTV is a non-pathogenic virus that is currently being explored as a marker of immune function in solid organ transplantation. Although TTV load at the time of the first vaccination was predictive of the future development of antibodies, we found that these levels did not correlate with the induction of T-cell responses or with the durability of either humoral or cellular responses.

We show that WLs, an overlooked group until now, have reduced humoral and cellular responses compared with controls. These patients had both reduced S-specific binding and neutralizing antibody responses at 28 days after vaccination, as well as reduced T-cell responses. At 6 months, the reduction in neutralizing antibodies and the decay in cellular responses proved disproportionately large in WLs compared with controls. This is important information, as WLs were not specifically investigated for COVID-19 vaccination responses before. Unfortunately, we did not include enough participants to determine associations between patient characteristics and the observed effects. With 33% of WLs in our study receiving corticosteroids, one could hypothesize that these drugs are the cause of the inferior responses and reduced durability. However, only one of the investigated markers, IGRA response at T3, was associated with the use of corticosteroids. Other factors, such as underlying illness and the presence of chronic lung disease, likely contribute further to the decreased development and rapid decline of immune responses.

This study has several limitations. We used two methods to measure T-cell responses, an interferon-γ release assay and an interferon-γ ELISpot. Both methods have specific advantages and disadvantages. The IGRA test was shown to be less sensitive in a cohort of KTRs (31), but the test provides reproducible results. The ELISpot has to be performed by the simultaneous testing of samples of interest because the method relies on isolation of viable lymphocytes from peripheral blood which may not have the same yield every time, leading to intertest variability. The ELISpot test is therefore especially suitable for determining changes in the numbers of reactive T cells. In our study, we were not able to determine the decay of T-cell responses for all cellular responders at the 28-day time point because of insufficient viable cells in some of the stored blood samples. Another limitation of this study is that nearly all LTRs included in this study received homologous antirejection regimens, not allowing for the differentiation between specific medication and immune responses. The strengths of this study include the prospective design and the inclusion of a large number of LTRs as well as WLs which allowed us to study for the first time both humoral and cellular responses in these patient populations with a 6-month follow-up.

Several studies have shown that LTRs have poor immune responses following vaccination against COVID-19. However, our results are encouraging as they suggest that humoral and cellular responses in LTRs, if they develop, decrease at rates comparable with controls. Our findings in the WL group are disconcerting. The initial development of antibodies in WLs may have given the impression that this group responded analogous to controls. However, the inferior cellular responses and the rapid decay of both humoral and cellular responses in this group imply that WLs may not be protected adequately by two vaccinations. Our results suggest that repeat boostering may be necessary for WLs to induce protection that lasts beyond the months immediately post-transplantation. Our results additionally suggest that other patients, who do develop antibodies following two vaccinations, could still experience a disproportionate decay. More research is needed examining the durability of immune responses of patient groups with immunocompromising conditions, especially those receiving corticosteroids.

## Data availability statement

The raw data supporting the conclusions of this article will be made available by the authors, without undue reservation.

## Ethics statement

This study was approved by the medical ethical review boards of the University Medical Center Groningen and Erasmus University Medical Center and was registered in the Dutch clinical trials register under number NL9538. The studies were conducted in accordance with the local legislation and institutional requirements. The participants provided their written informed consent to participate in this study.

## Author contributions

RH: Data curation, Writing – original draft, Writing – review & editing. SL: Data curation, Formal analysis, Investigation, Writing – review & editing. CG: Conceptualization, Data curation, Formal analysis, Investigation, Methodology, Validation, Writing – review & editing. EV: Conceptualization, Investigation, Supervision, Writing – review & editing. JMV: Formal analysis, Software, Validation, Writing – review & editing. MH: Investigation, Supervision, Writing – review & editing. MK: Formal analysis, Supervision, Writing – review & editing. NW: Data curation, Investigation, Validation, Writing – review & editing. SB: Data curation, Investigation, Validation, Writing – review & editing. SS: Data curation, Investigation, Validation, Writing – review & editing. SR: Data curation, Investigation, Validation, Writing – review & editing. JvG: Investigation, Validation, Writing – review & editing. WS: Data curation, Project administration, Resources, Writing – review & editing. HN: Methodology, Project administration, Supervision, Validation, Writing – review & editing. DV: Conceptualization, Data curation, Investigation, Methodology, Writing – review & editing. RD: Conceptualization, Formal analysis, Investigation, Methodology, Supervision, Validation, Visualization, Writing – review & editing. CV: Conceptualization, Formal analysis, Funding acquisition, Investigation, Methodology, Project administration, Supervision, Validation, Writing – review & editing.
