# Supplementary material for: Humoral and cellular immune responses after COVID-19 vaccination of lung transplant recipients and patients on the waiting list: a 6-month follow-up
Source: Front Immunol. 2024 Jan 4;14:1254659. doi: 10.3389/fimmu.2023.1254659 (PMC10794507; doi:10.3389/fimmu.2023.1254659)
Supplement: Supplementary file 1 [file Table_1.docx]

**Supplementary data**

**Table S1a-b: Spike-specific IgG responses and kinetics in the three groups (Lung transplant recipients (LTR), Waitlist (WL) and Controls), during the six months after vaccination.**

**Table S1a:** Spike-specific IgG levels (BAU/ml) at 28 days after the first vaccination (T1), 28 days after the second vaccination (T2) and six months after the second vaccination (T3). IgG levels are shown overall, and in responders only (with responders defined as having IgG>33.8 BAU/ml).

|  | 28 days after 1st vaccination (T1) | | | | 28 days after second vaccination (T2) | | | | 6 months after second vaccination (T3) | | | |
| --- | --- | --- | --- | --- | --- | --- | --- | --- | --- | --- | --- | --- |
|  | N | IgG ≥ 33.8 BAU/ml | IgG level in all (BAU/ml) | IgG (BAU/ml) in responders only | N | IgG ≥ 33.8 BAU/ml | IgG level in all (BAU/ml) | IgG (BAU/ml) in responders only | N | IgG ≥ 33.8 BAU/ml | IgG level in all (BAU/ml) | IgG (BAU/ml) in responders only |
|  |  | N (%) | Median (IQR) | Median (IQR) |  | N (%) | Median (IQR) | Median (IQR) |  | N (%) | Median (IQR) | Median (IQR) |
|  |  |  |  |  |  |  |  |  |  |  |  |  |
| LTR | 102 | 4 (3.9) | 5 (5-5) | 77 (54-88) | 102 | 21 (20.6) | 5 (5-25) | 311 (96-829) | 99 | 13 (13.1) | 5 (5-15) | 85 (50-242) |
| WL | 58 | 51 (87.9) | 249 (84-565) | 325 (120-640) | 55 | 55 (100) | 2520 (1520-4340) | 2520 (1520-4340) | 44 | 40 (90.9) | 267 (90-753) | 320 (145-956) |
| Controls | 61 | 59 (96.7) | 562 (333-1075) | 627 (364-1090) | 60 | 60 (100) | 3220 (2160-5593) | 3220 (2160-5593) | 55 | 55 (100) | 465 (309-951) | 465 (309-951) |
|  |  |  |  |  |  |  |  |  |  |  |  |  |
| Between groups p-values |  |  |  |  |  |  |  |  |  |  |  |  |
| LTR vs Controls |  | 5.30*10-32 | 6.93*10-30 | 8.81*10-4 |  | 1.64*10-22 | 5.43*10-25 | 7.64*10-9 |  | 2.43*10-25 | 7.78*10-25 | 2.0*10-5 |
| WL vs Controls |  | 0.090 | 4.5*10-5 | 2.59*10-4 |  | NA | 0.023 | 0.023 |  | 0.036 | 1.08*10-3 | 0.017 |
| LTR vs WL |  | 5.59*10-27 | 6.80*10-27 | 0.010 |  | 2.12*10-21 | 1.44*10-23 | 7.48*10-7 |  | 6.22*10-19 | 1.33*10-19 | 8.14*10-3 |

**Table S1b:** Median (IQR) of percentage decline in Spike-specific IgG between 28 days and six months after second vaccination (only in responders, i.e. those with IgG ≥ 33.8 BAU at 28 days after second vaccination)

|  | N | Median (IQR) | Within groups  P-values 28 days versus 6 months |
| --- | --- | --- | --- |
| LTR | 21 | -70.9 (-95.0 - -56.8) | 7.97*10-5 |
| WL | 43 | -86.7 (-96.9 - -69.5) | 1.16*10-8 |
| Controls | 51 | -85.2 (-89.8 - -78.4) | 5.15*10-10 |
|  |  |  |  |
| Between groups p-values |  |  |  |
| LTR vs Controls |  | 0.247 |  |
| WL vs Controls |  | 0.329 |  |
| LTR vs WL |  | 0.211 |  |

**Table S2a-b: PRNT50 responses and kinetics in the three groups (LTR, WL and Controls), during the six months after the second vaccination, expressed in geometric mean titer (GMT) of neutralizing antibodies.**

**Table S2a:** PRNT50 responses at 28 days after the second vaccination (T2) and six months after the second vaccination (T3).

PRNT50 levels are shown overall, and in responders only (with responders defined as having PRNT50 >10).

|  | 28 days after second vaccination | | | | 6 months after second vaccination | | | |
| --- | --- | --- | --- | --- | --- | --- | --- | --- |
|  | N | PRNT50 > 10 | PRNT50 GMT in all | PRNT50 GMT in responders | N | PRNT50 > 10 | PRNT50 GMT in all | PRNT50 GMT in responders |
|  |  | N (%) | GMT (%sd) | GMT (%sd) |  | N (%) | GMT (%sd) | GMT (%sd) |
|  |  |  |  |  |  |  |  |  |
| LTR | 21 | 15 (71.4) | 40.42 (3.90) | 70.66 (3.37) | 14 | 7 (50.0) | 18.83 (2.11) | 35.45 (1.70) |
| WL | 55 | 54 (98.2) | 217.32 (2.64) | 230.07 (2.42) | 39 | 28 (71.8) | 33.53 (2.86) | 53.94 (2.34) |
| Controls | 42 | 42 (100) | 453.76 (2.13) | 453.76 (2.13) | 41 | 40 (97.6) | 122.12 (2.57) | 130.00 (2.37) |
|  |  |  |  |  |  |  |  |  |
| Between groups p-values |  |  |  |  |  |  |  |  |
| LTR vs Controls |  | 7.99*10-4 | 4.36*10-8 | 2.78*10-5 |  | 1.18*10-4 | 1.29*10-8 | 3.93*10-4 |
| WL vs Controls |  | 1.00 | 1.01*10-4 | 1.37*10-4 |  | 1.16*10-3 | 1.36*10-7 | 9.44*10-5 |
| LTR vs WL |  | 1.42*10-3 | 1.65*10-5 | 7.91*10-5 |  | 0.191 | 0.065 | 0.223 |

**Table S2b:** Median (IQR) of percentage decline in PRNT50 between 28 days and six months after the second vaccination (only in those with PRNT50 > 10 at 28 days after second vaccination)

|  | N | Median (IQR) | Within groups  P-values 28d vs 6mo |
| --- | --- | --- | --- |
| LTR | 11 | -71.4 (-85.0 - -47.4) | 0.021 |
| WL | 39 | -89.0 (-93.2 - -80.1) | 6.64*10-8 |
| Controls | 41 | -75.3 (-85.4 - -46.0) | 4.54*10-8 |
|  |  |  |  |
| Between groups p-values |  |  |  |
| LTR vs Controls |  | 0.599 |  |
| WL vs Controls |  | 5.73*10-5 |  |
| LTR vs WL |  | 0.013 |  |

**Table S3a-b: ELIspot response and kinetics in the three groups (LTR, WL and Controls), during the six months after vaccination.**

**Table S3a:** ELIspot, with responses measured in number of spot forming colonies (SFC)/ 10^6^ PBMC, and in number (%) responders defined as >50 SFC/10^6^ PBMC AND minimum 2-fold increase from baseline.

|  |  | 28 days after second vaccination | | |
| --- | --- | --- | --- | --- |
|  | N | ELISPOT ≥ 50* | ELISPOT level in all | ELISPOT level in responders |
|  |  | N (%) | Median (IQR) | Median (IQR) |
|  |  |  |  |  |
| LTR | 95 | 28 (29.5) | 38 (10-75) | 124 (71-155) |
| WL | 49 | 27 (55.1) | 100 (33-243) | 232 (103-318) |
| Controls | 23 | 18 (78.3) | 132 (55-193) | 152 (103-292) |
|  |  |  |  |  |
| Between groups  p-values |  |  |  |  |
| LTR vs Controls |  | 1.67*10-5 | 8.90*10-5 | 0.083 |
| WL vs Controls |  | 0.058 | 0.534 | 0.379 |
| LTR vs WL |  | 2.71*10-3 | 2.27*10-4 | 4.80*10-3 |

* & foldchange vs pre-vaccination ≥ 2

**Table S3b**: Decline in Elispot response between 28 days and six months after second vaccination in the three groups.

|  |  | 28 days after second vaccination |  | 6 months after second vaccination |  | percentage decline in ELISPOT between 28 days and 6 months after second vaccination |  |
| --- | --- | --- | --- | --- | --- | --- | --- |
|  | N | ELISPOT level in all | N | ELISPOT level in all |  |  |  |
|  |  | Median (IQR) |  | Median (IQR) |  | Median (IQR) | Within groups P-values 28d vs 6mo |
|  |  |  |  |  |  |  |  |
| LTR | 12 | 216 (90-348) | 12 | 133 (20-285) |  | -36.8 (-93.1 – 35.0) | 0.209 |
| WL | 15 | 342 (192-572) | 15 | 60 (30-376) |  | -74.1 (-85.4 - -54.6) | 9.82*10-4 |
| Controls | 19 | 573 (250-832) | 19 | 293 (103-452) |  | -49.4 (-70.3 - -36.7) | 9.67*10-4 |
|  |  |  |  |  |  |  |  |
| Between groups  p-values |  |  |  |  |  |  |  |
| LTR vs Contols |  | 0.012 |  | 0.152 |  | 0.484 |  |
| WL vs Conrols |  | 0.089 |  | 0.027 |  | 0.025 |  |
| LTR vs WL |  | 0.167 |  | 0.867 |  | 0.126 |  |

**Table S4a-b: IGRA-responses and kinetics, in the three groups (LTR, WL and Controls), during the six months after the second vaccination.**

**Table S4a:** IGRA responses at 28 days and at six months after the second vaccination, measured as Ag2-levels are shown overall and in responders only (with responders defined as having Ag2 >0.15 IU/ml).

|  |  | 28 days after second vaccination | | |  | 6 months after second vaccination | | |
| --- | --- | --- | --- | --- | --- | --- | --- | --- |
|  | N | IGRA_Ag2 ≥ 0.15 | IGRA_Ag2 level in all | IGRA_Ag2 level in responders | N | IGRA_Ag2 ≥ 0.15 | IGRA_Ag2 level in all | IGRA_Ag2 level in responders |
|  |  | N (%) |  | Median (IQR) |  | N (%) |  | Median (IQR) |
|  |  |  |  |  |  |  |  |  |
| LTR | 100 | 12 (12.0) | 0.04 (0.01-0.06) | 0.27 (0.18-0.35) | 91 | 9 (9.9) | 0.02 (0.01-0.06) | 0.40 (0.18-0.73) |
| WL | 55 | 30 (54.5) | 0.18 (0.07-0.74) | 0.61 (0.40-1.09) | 40 | 16 (40.0) | 0.06 (0.02-0.25) | 0.28 (0.21-0.46) |
| Controls | 46 | 42 (91.3) | 1.07 (0.52-2.20) | 1.19 (0.68-2.41) | 41 | 32 (78.0) | 0.78 (0.24-1.75) | 1.00 (0.68-1.90) |
|  |  |  |  |  |  |  |  |  |
| Between groups p-values |  |  |  |  |  |  |  |  |
| LTR vs Controls |  | 2.95*10-20 | 1.66*10-19 | 2.89*10-5 |  | 4.85*10-15 | 1.72*10-16 | 3.07*10-3 |
| WL vs Controls |  | 4.77*10-5 | 1.21*10-7 | 6.93*10-4 |  | 4.93*10-4 | 8.00*10-8 | 3.57*10-5 |
| LTR vs WL |  | 1.18*10-8 | 2.71*10-11 | 4.08*10-3 |  | 5.37*10-5 | 1.50*10-4 | 0.777 |

**Table S4b:** Median (IQR) of percentage decline in IGRA Ag2-levels, between 28 days and six months second vaccination (only in those with Ag2 levels ≥ 0.15 at 28 days after second vaccination)

|  | N | Median (IQR) | Within groups P-values 28d days vs 6 months |
| --- | --- | --- | --- |
| LTR | 11 | -18.3 (-61.6 - 75.0) | 0.790 |
| WL | 19 | -71.4 (-88.0 - -27.3) | 1.87*10-4 |
| Controls | 37 | -40.2 (-63.5 - -8.0) | 9.63*10-5 |
|  |  |  |  |
| Between groups p-values |  |  |  |
| LTR vs Controls |  | 0.454 |  |
| WL vs Controls |  | 0.040 |  |
| LTR vs WL |  | 0.064 |  |

**Table S5a-f: Factors associated with Spike-specific IgG responses in the three groups (LTR, WL and Controls), during the six months after vaccination.**

**Table S5a and b**: Factors associated with IgG response at 28 days after the second vaccination, analyzed by logistic regression.

**S5a:** Univariate analysis

|  | LTR | | | WL | | Controls | |
| --- | --- | --- | --- | --- | --- | --- | --- |
|  | OR | 95% CI | P | OR | P | OR | P |
| Female gender | 0.808 | 0.307 – 2.126 | 0.665 | NA |  | NA |  |
| Age | 0.962 | 0.929 – 0.996 | 0.028 | NA |  | NA |  |
| TTV | 0.646 | 0.492 – 0.847 | 0.002 | NA |  | NA |  |
| Time since Tx > 100 months | 2.287 | 0.841 – 6.224 | 0.105 | NA |  | NA |  |
| Azathioprine | 6.016 | 1.453 – 24.907 | 0.013 | NA |  | NA |  |
| MMF | 0.132 | 0.037 – 0.474 | 0.002 | NA |  | NA |  |
| Prednisolone | 0.395 | 0.086 – 1.806 | 0.231 | NA |  | NA |  |

**S5b:** Multivariate analysis (All variables with p < 0.15 in univariate model. N.b., Azathioprin and MMF are highly correlated and cannot be in the same model):

|  | LTR | | | WL | | Controls | |
| --- | --- | --- | --- | --- | --- | --- | --- |
|  | OR | 95% CI | P | OR | P | OR | P |
| Female gender |  |  |  | NA |  | NA |  |
| Age | 0.954 | 0.917 – 0.993 | 0.023 | NA |  | NA |  |
| TTV | 0.610 | 0.445 – 0.838 | 0.002 | NA |  | NA |  |
| Time since Tx > 100 months | 1.263 | 0.355 – 4.494 | 0.718 | NA |  | NA |  |
| Azathioprine |  |  |  | NA |  | NA |  |
| MMF | 0.107 | 0.022 – 0.507 | 0.005 | NA |  | NA |  |
| Prednisolone |  |  |  | NA |  | NA |  |

**Table S5c and d**: Factors associated with IgG response in the three groups (LTR, Waitlist and Controls), at six months after the second vaccination, analyzed by logistic regression.

**Table S5c:** Univariate analysis

|  | LTR | | | Waitlist | | | Controls | |
| --- | --- | --- | --- | --- | --- | --- | --- | --- |
|  | OR | 95% CI | P | OR | 95% CI | P | OR | P |
| Female gender | 1.406 | 0.436 – 4.531 | 0.568 | 1.500 | 0.191 – 11.763 | 0.700 | NA |  |
| Age | 0.959 | 0.921 – 0.998 | 0.042 | 0.800 | 0.608 – 1.052 | 0.110 | NA |  |
| TTV | 0.840 | 0.632 – 1.115 | 0.228 | 1.044 | 0.450 – 2.420 | 0.920 | NA |  |
| Time since Tx > 100 months | 3.611 | 1.092 – 11.945 | 0.035 | NA |  |  | NA |  |
| Azathioprine | 0.813 | 0.093 – 7.087 | 0.851 | NA |  |  | NA |  |
| MMF | 0.390 | 0.090 – 1.683 | 0.207 | NA |  |  | NA |  |
| Prednisolone | 0.900 | 0.100 – 8.141 | 0.925 | 0.160 | 0.015 – 1.696 | 0.128 | NA |  |

**Table S5d:** Multivariate (All variables with p < 0.15 in univariate model):

|  | LTR | | | Waitlist | | | Controls | |
| --- | --- | --- | --- | --- | --- | --- | --- | --- |
|  | OR | 95% CI | P | OR | 95% CI | P | OR | P |
| Female gender |  |  |  |  |  |  | NA |  |
| Age | 0.951 | 0.912 – 0.992 | 0.020 | 0.728 | 0.490 – 1.080 | 0.115 | NA |  |
| TTV |  |  |  |  |  |  | NA |  |
| Time since Tx > 100 months | 4.791 | 1.320 – 17.390 | 0.017 |  |  |  | NA |  |
| Azathioprine |  |  |  |  |  |  | NA |  |
| MMF |  |  |  |  |  |  | NA |  |
| Prednisolone |  |  |  | 0.107 | 0.007 – 1.729 | 0.116 | NA |  |

**Table S5e and f:** Factors associated with S-specific IgG decline between 28 days and six months after the second vaccination in the three groups (LTR, Waitlist and Controls), analyzed by linear regression

**Table S5e:** Univariate analysis

|  | LTR | | | Waitlist | | | Controls | | |
| --- | --- | --- | --- | --- | --- | --- | --- | --- | --- |
|  | B | 95% CI | P | B | 95% CI | P | B | 95% CI | P |
| Female gender | -14.7 | -45.1 – 15.6 | 0.322 | -12.2 | -31.5 – 7.0 | 0.207 | -7.5 | -13.6 - -1.4 | 0.018 |
| Age | 0.09 | -.98 – 1.16 | 0.860 | -0.34 | -1.19 – 0.52 | 0.431 | -0.19 | -0.40 – 0.02 | 0.070 |
| TTV | 5.58 | -2.86 – 14.02 | 0.183 | 6.06 | -2.08 – 14.20 | 0.140 | NA |  |  |
| Time since Tx > 100 months | -20.0 | -49.6 – 9.6 | 0.174 | NA |  |  | NA |  |  |
| Azathioprine | -23.3 | -57.7 – 11.2 | 0.174 | NA |  |  | NA |  |  |
| MMF | 27.3 | -2.7 – 57.3 | 0.072 | NA |  |  | NA |  |  |
| Prednisolone | 18.5 | -24.7 – 61.6 | 0.381 | -10.3 | -30.2 – 9.5 | 0.300 | NA |  |  |

**Table S5f:** Multivariate analysis (All variables with p < 0.20 in univariate model. NB, Azathioprin and MMF are highly correlated and cannot be in the same model):

|  | LTR | | | Waitlist | | | Controls | | |
| --- | --- | --- | --- | --- | --- | --- | --- | --- | --- |
|  | B | 95% CI | P | B | 95% CI | P | B | 95% CI | P |
| Female gender |  |  |  |  |  |  | -6.53 | -12.74 - -0.323 | 0.040 |
| Age |  |  |  |  |  |  | -0.15 | -0.354 – 0.062 | 0.166 |
| TTV | 4.46 | -5.11 – 14.03 | 0.339 | 6.06 | -2.08 – 14.20 | 0.140 |  |  |  |
| Time since Tx > 100 months | -2.87 | -39.54 – 33.81 | 0.871 |  |  |  |  |  |  |
| Azathioprine |  |  |  |  |  |  |  |  |  |
| MMF | 24.26 | -9.48 – 58.00 | 0.148 |  |  |  |  |  |  |
| Prednisolone |  |  |  |  |  |  |  |  |  |

**Table S6 a-f: Factors associated with PRNT50 response in the three groups (LTR, Waitlist and Controls), during the six months after vaccination.**

**Table S6 a-b:** Factors associated with PRNT50 response at 28 days after the second vaccination, analyzed by logistic regression

**Table S6a**: Univariate analysis

|  | LTR | | | Waitlist | | Controls | |
| --- | --- | --- | --- | --- | --- | --- | --- |
|  | OR | 95% CI | P | OR | P | OR | P |
| Female gender | 5.714 | 0.532 – 61.410 | 0.150 | NA |  | NA |  |
| Age | 0.937 | 0.863 – 1.019 | 0.127 | NA |  | NA |  |
| TTV | 0.739 | 0.408 – 1.337 | 0.317 | NA |  | NA |  |
| Time since Tx > 100 months | 1.750 | 0.242 – 12.642 | 0.579 | NA |  | NA |  |
| Azathioprine | 0.500 | 0.060 – 4.153 | 0.521 | NA |  | NA |  |
| MMF | 1.000 | 0.134 – 7.451 | 1.000 | NA |  | NA |  |
| Prednisolone | 1.300 | 0.095 – 17.726 | 0.844 | NA |  | NA |  |

**Table S6b:** Multivariate analysis (All variables with p < 0.15 in univariate model):

|  | LTR | | | Waitlist | | Controls | |
| --- | --- | --- | --- | --- | --- | --- | --- |
|  | OR | 95% CI | P | OR | P | OR | P |
| Female gender | 3.999 | 0.337 – 47.518 | 0.272 | NA |  | NA |  |
| Age | 0.945 | 0.864 – 1.034 | 0.220 | NA |  | NA |  |

**Table S6c and d:** Factors associated with PRNT50 levels at six months after the second vaccination, analyzed by logistic regression.

**Table S6c:** Univariate analysis

|  | LTR | | | Waitlist | | | Controls | |
| --- | --- | --- | --- | --- | --- | --- | --- | --- |
|  | OR | 95% CI | P | OR | 95% CI | P | OR | P |
| Female gender | 15.000 | 1.031 – 218.300 | 0.047 | 0.500 | 0.109 – 2.294 | 0.373 | NA |  |
| Age | 0.990 | 0.918 – 1.068 | 0.803 | 0.983 | 0.919 – 1.052 | 0.625 | NA |  |
| TTV | 0.886 | 0.487 – 1.613 | 0.692 | 2.563 | 0.965 – 6.807 | 0.059 | NA |  |
| Time since Tx > 100 months | 15.000 | 1.031 – 218.300 | 0.047 | NA |  |  | NA |  |
| Azathioprine | NA |  |  | NA |  |  | NA |  |
| MMF | 0.417 | 0.029 – 6.064 | 0.522 | NA |  |  | NA |  |
| Prednisolone | 1.000 | 0.050 – 19.963 | 1.000 | 0.700 | 0.160 – 3.066 | 0.636 | NA |  |

**Table 6d:** Multivariate analysis (All variables with p < 0.15 in univariate model):

|  | LTR | | | Waitlist | | | Controls | |
| --- | --- | --- | --- | --- | --- | --- | --- | --- |
|  | OR | 95% CI | P | OR | 95% CI | P | OR | P |
| Female gender | 10.642 | 0.527 – 214.893 | 0.123 |  |  |  | NA |  |
| TTV |  |  |  | 2.563 | 0.965 – 6.807 | 0.059 | NA |  |
| Time since Tx > 100 months | 10.642 | 0.527 – 214.893 | 0.123 |  |  |  | NA |  |

**Table S6e-f:** Factors associated with percentage decline in PRNT50 between 28 days and six months after second vaccination (only in those with PRNT50 > 10 at 28 days after second vaccination), analyzed with linear regression.

**Table S6e:** Univariate analysis

|  | LTR | | | Waitlist | | | Controls | | |
| --- | --- | --- | --- | --- | --- | --- | --- | --- | --- |
|  | B | 95% CI | P | B | 95% CI | P | B | 95% CI | P |
| Female gender | 49.11 | -60.77 – 159.00 | 0.338 | -10.62 | -31.27 – 10.04 | 0.304 | -7.51 | -27.96 – 12.94 | 0.462 |
| Age | 2.23 | -1.41 – 5.87 | 0.198 | -0.008 | -0.91 – 0.89 | 0.985 | -0.96 | -1.58 - -0.35 | 0.003 |
| TTV | 0.82 | -33.49 – 35.13 | 0.958 | 9.17 | 0.83 – 17.51 | 0.032 | NA |  |  |
| Time since Tx > 100 months | 18.61 | -96.49 – 133.72 | 0.723 | NA |  |  | NA |  |  |
| Azathioprine | -24.24 | -224.25 – 175.78 | 0.790 | NA |  |  | NA |  |  |
| MMF | 42.42 | -83.22 – 168.05 | 0.465 | NA |  |  | NA |  |  |
| Prednisolone | 48.33 | -96.87 – 193.53 | 0.471 | 7.36 | -14.59 – 29.31 | 0.501 | NA |  |  |

**Table S7a-c: Factors associated with ELIspot response in the three groups (LTR, Waitlist and Controls), during the six months after vaccination.**

**Table S7a-b:** Factors associated with ELIspot response at 28 days after second vaccination, analyzed by logistic regression.

**Table S7a:** Univariate analysis:

|  | LoTX | | | WL | | | C | | |
| --- | --- | --- | --- | --- | --- | --- | --- | --- | --- |
|  | OR | 95% CI | P | OR | 95% CI | P | OR | 95% CI | P |
| Female gender | 0.948 | 0.392 – 2.294 | 0.906 | 2.000 | 0.629 – 6.361 | 0.240 | 1.333 | 0.173 – 10.254 | 0.782 |
| Age | 0.999 | 0.967 – 1.033 | 0.971 | 0.950 | 0.883 – 1.021 | 0.162 | 1.010 | 0.939 – 1.085 | 0.794 |
| TTV | 0.887 | 0.717 – 1.097 | 0.267 | 0.853 | 0.537 – 1.355 | 0.502 | NA |  |  |
| Time since Tx > 100 months | 5.538 | 2.115 – 14.504 | 0.000 | NA |  |  | NA |  |  |
| Azathioprin | 0.782 | 0.148 – 4.133 | 0.772 | NA |  |  | NA |  |  |
| MMF | 1.293 | 0.323 – 5.182 | 0.717 | NA |  |  | NA |  |  |
| Prednisolone | 0.281 | 0.059 – 1.350 | 0.113 | 0.420 | 0.126 – 1.397 | 0.157 | NA |  |  |

**Table S7b:** Multivariate analysis (All variables with p < 0.15 in univariate model):

|  | LoTX | | | WL | | | C | | |
| --- | --- | --- | --- | --- | --- | --- | --- | --- | --- |
|  | OR | 95% CI | P | OR | 95% CI | P | OR | 95% CI | P |
| Time since Tx > 100 months | 5.157 | 1.875 – 14.181 | 0.001 |  |  |  |  |  |  |
| Prednisolone | 0.681 | 0.123 – 3.773 | 0.660 |  |  |  |  |  |  |

**Table S7c:** Factors associated with percentage decline in ELIspot responses between 28 days and six months after the second vaccination (only in those with paired ELIspot), analyzed by linear regression (univariate).

|  | LTR | | | Waitlist | | | Controls | | |
| --- | --- | --- | --- | --- | --- | --- | --- | --- | --- |
|  | B | 95% CI | P | B | 95% CI | P | B | 95% CI | P |
| Female gender | 35.06 | -69.04 – 139.16 | 0.470 | 25.82 | -2.00 – 53.64 | 0.066 | -28.29 | -73.81 – 17.23 | 0.207 |
| Age | -2.52 | -5.12 – 0.08 | 0.056 | -0.49 | -1.80 – 0.82 | 0.434 | 0.99 | -0.88 – 2.85 | 0.279 |
| TTV | 8.24 | -25.64 – 42.12 | 0.600 | -5.98 | -17.86 – 5.90 | 0.294 | NA |  |  |
| Time since LTX > 100 months | -1.67 | -103.96 – 100.63 | 0.972 | NA |  |  | NA |  |  |
| Azathioprine | 78.41 | -95.51 – 252.329 | 0.339 | NA |  |  | NA |  |  |
| MMF | -51.13 | -181.58 – 79.33 | 0.403 | NA |  |  | NA |  |  |
| Prednisolone | 37.90 | -75.48 – 151.27 | 0.474 | -7.37 | -37.68 – 22.94 | 0.608 | NA |  |  |

**Table S8 a-e: Factors associated with IGRA response in the three groups (LTR, Waitlist and Controls), over six months after second vaccination.**

**Table S8 a and b**: Factors associated with IGRA responses, at 28 days after second vaccination, analyzed by logistic regression on those with IGRA Ag2-levels ≥ 0.15 at 28 days after second vaccination.

**Table S8a:** Univariate analysis

|  | LTR | | | Waitlist | | | Controls | | |
| --- | --- | --- | --- | --- | --- | --- | --- | --- | --- |
|  | OR | 95% CI | P | OR | 95% CI | P | OR | 95% CI | P |
| Female gender | 3.947 | 1.000 – 15.582 | 0.050 | 2.167 | 0.727 – 6.455 | 0.165 | 7.250 | 0.684 – 76.865 | 0.100 |
| Age | 0.984 | 0.942 – 1.026 | 0.984 | 0.998 | 0.949 – 1.049 | 0.931 | 0.964 | 0.885 – 1.049 | 0.393 |
| TTV | 1.167 | 0.864 – 1.576 | 0.313 | 0.984 | 0.628 – 1.541 | 0.944 | NA |  |  |
| Time since Tx > 100 months | 0.840 | 0.210 – 3.360 | 0.805 | NA |  |  | NA |  |  |
| Azathioprine | 0.909 | 0.104 – 7.980 | 0.931 | NA |  |  | NA |  |  |
| MMF | 1.571 | 0.184 – 13.388 | 0.679 | NA |  |  | NA |  |  |
| Prednisolone | NA |  |  | 0.271 | 0.082 – 0.890 | 0.031 | NA |  |  |

**Table S8 c-d:** Factors associated with IGRA responses in the three groups (LTR, Waitlist and Controls), at six months after second vaccination, analyzed by logistic regression.

**Table S8c:** Univariate analysis:

|  | LTR | | | Waitlist | | | Controls | | |
| --- | --- | --- | --- | --- | --- | --- | --- | --- | --- |
|  | OR | 95% CI | P | OR | 95% CI | P | OR | 95% CI | P |
| Female gender | 2.686 | 0.628 – 11.488 | 0.183 | 1.320 | 0.345 – 5.050 | 0.685 | 2.750 | 0.606 – 12.479 | 0.190 |
| Age | 0.985 | 0.937 – 1.035 | 0.551 | 1.001 | 0.946 – 1.059 | 0.968 | 0.975 | 0.922 – 1.030 | 0.367 |
| TTV | 1.033 | 0.742 – 1.439 | 0.848 | 0.879 | 0.503 – 1.535 | 0.650 | NA |  |  |
| Time since Tx > 100 months | 1.452 | 0.333 – 6.329 | 0.619 | NA |  |  | NA |  |  |
| Azathioprine | NA |  |  | NA |  |  | NA |  |  |
| MMF | NA |  |  | NA |  |  | NA |  |  |
| Prednisolone | NA |  |  | 0.143 | 0.027 – 0.769 | 0.024 | NA |  |  |

**Table S8d:** Multivariate analysis (All variables with p < 0.15 in univariate model):

|  | LTR | | | Waitlist | | | Controls | | |
| --- | --- | --- | --- | --- | --- | --- | --- | --- | --- |
|  | OR | 95% CI | P | OR | 95% CI | P | OR | 95% CI | P |
| Prednisolone |  |  |  | 0.143 | 0.027 – 0.769 | 0.024 |  |  |  |

**Table S8e:** Factors associated with percentage decline in IGRA Ag2-levels between 28 days and six months after the second vaccination (only in those with IGRA Ag2 levels ≥ 0.15 at 28 days after the second vaccination), analyzed by linear regression

Univariate analysis:

|  | LTR | | | Waitlist | | | Controls | | |
| --- | --- | --- | --- | --- | --- | --- | --- | --- | --- |
|  | B | 95% CI | P | B | 95% CI | P | B | 95% CI | P |
| Female gender | 31.95 | -201.45 – 265.35 | 0.764 | -9.07 | -53.94 – 35.80 | 0.675 | 26.58 | -22.63 – 75.79 | 0.280 |
| Age | 1.80 | -5.79 – 9.39 | 0.604 | 0.24 | -1.24 – 1.72 | 0.735 | 1.15 | -0.47 – 2.76 | 0.159 |
| TTV | 12.16 | -42.05 – 66.37 | 0.624 | 7.28 | -10.04 – 24.60 | 0.386 | NA |  |  |
| Time since Tx > 100 months | -67.35 | -73.93 – 165.34 | 0.523 | NA |  |  | NA |  |  |
| Azathioprine | -128.69 | -479.01 – 221.62 | 0.427 | NA |  |  | NA |  |  |
| MMF | 128.69 | -221.62 – 479.01 | 0.427 | NA |  |  | NA |  |  |
| Prednisolone | NA |  |  | 21.39 | -22.37 – 65.15 | 0.317 | NA |  |  |
